# Allogeneic CD19/CD22 CAR T-Cell Therapy for B-Cell Acute Lymphoblastic Leukemia

**DOI:** 10.1001/jamaoncol.2024.0473

**Published:** 2024-04-18

**Authors:** Laurent Phely, Luca Hensen, Christoph Faul, Christer Alexander Ruff, Dina Schneider, Wolfgang Andreas Bethge, Claudia Lengerke

**Affiliations:** 1 Department of Internal Medicine II, Hematology, Oncology, Clinical Immunology and Rheumatology, University Hospital Tuebingen, Tuebingen, Germany; 2 Department of Diagnostic and Interventional Neuroradiology, University Hospital Tubingen, Tubingen, Germany; 3 Miltenyi Biotec, Gaithersburg, Maryland

## Abstract

This case series reports durable remissions in 2 patients with relapsed/refractory B-cell acute lymphoblastic leukemia treated with allogeneic bispecific CD19/CD22-targeting chimeric antigen receptor T cells.

Antibodies, T-cell engagers, immunotoxins, or chimeric antigen receptor (CAR) T cells targeting B-cell antigens have revolutionized the treatment of B-cell acute lymphoblastic leukemia (ALL). CD19-targeting CAR T cells induce remission in approximately 70% of adults with relapsed/refractory B-cell ALL, but approximately 30% to 60% relapse due to loss of CD19 expression on tumor cells and/or limited CAR T-cell persistence.^1,2^ Bispecific constructs cotargeting CD19 and the alternative B-cell antigen CD22^3,4^ may reduce antigen escape. The use of allogeneic leukocytes may improve CAR T-cell functionality. Herein, we report durable remissions in 2 patients with relapsed/refractory B-cell ALL treated with allogeneic bispecific CD19/CD22-targeting CAR T cells.

## Methods

Patients’ characteristics and workup are summarized in the eMethods in Supplement 1. CAR T-cell treatments were discussed in the University Hospital Tübingen Cell Therapy Board and performed with the patients’ written informed consent based on hospital exemption for advanced therapy medicinal products treatment and declaration to the competent authority, Paul-Ehrlich-Institut, according to the section 67 of the German Medicines Act.

Leukocytes from the patient or the hematopoietic cell donor were transduced with a bispecific human anti-CD19/anti-CD22 lentiviral construct (Miltenyi Biotec), expanded in the CliniMACS Prodigy (Miltenyi Biotec) in the Good Manufacturing Practice Laboratory at the University Hospital Tuebingen, and infused (day 0) after lymphodepletion with fludarabine, 25 mg/m^2^ (day −5 until day −3), and cyclophosphamide, 1000 mg/m^2^ (day −3).^5^ CAR detection reagent (Miltenyi Biotec), MACSQuant (Miltenyi Biotec), and FACSLyric Flow Cytometer (BD Biosciences) were used for CAR T-cell quantification between January 2020 and September 2023.

## Results

**Figure 1.**
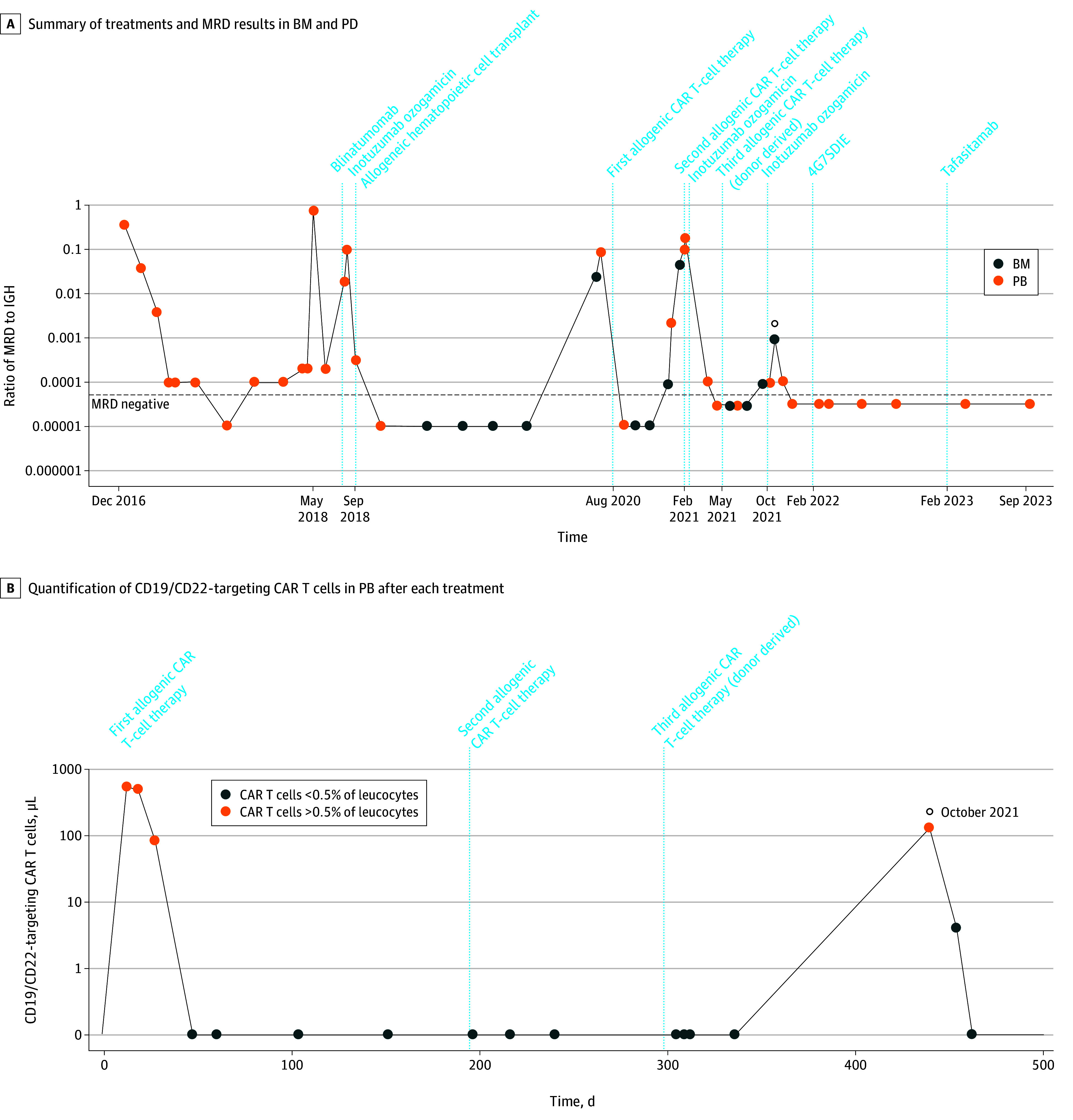
Treatment Response and Chimeric Antigen Receptor (CAR) T-Cell Quantification in Patient 1 A, Summary of treatments and minimal residual disease (MRD) results in bone marrow (BM) and peripheral blood (PB) (*IGHV3-21* and *D3-16 J8* along logarithmic scale; MRD negativity defined as <1 × 10^4^, represented by the horizontal dashed line; the open circle marks a rise in MRD). B, Quantification of CD19/CD22-targeting CAR T cells in PB after each CAR T-cell treatment (the open circle marks CAR T-cell expansion at the rise of MRD).

A woman in her late 50s with B-cell ALL relapse after receiving chemotherapy, blinatumomab, inotuzumab ozogamicin, and allogeneic hematopoietic cell transplant (alloHCT) was treated with 3 × 10^6^/kg bodyweight patient-derived fresh allogeneic bispecific CD19/CD22-targeting CAR T cells (Figure 1A). CAR T cells expanded, inducing IL-6 and IL-2 receptors (Figure 1B), but then dropped, allowing CD19^+^/CD22^+^ relapse. Reinfused cryopreserved CAR T cells (3 × 10^6^/kg bodyweight) failed to expand and to induce antileukemic effects. Next, fresh allogeneic CD19/CD22-targeting CAR T cells were manufactured from mononuclear cells of the healthy 8/10 HLA-matched unrelated hematopoietic cell donor and applied after bridging with inotuzumab ozogamicin. Maintenance with inotuzumab ozogamicin, 4G7SDIE, and tafasitamab was given for the multiple relapses. Rise in minimal residual disease triggered CAR T-cell expansion, suggesting active CAR T-cell immune surveillance (Figure 1). Sustained remission was documented approximately 3 years after the third CAR T-cell therapy.

**Figure 2.**
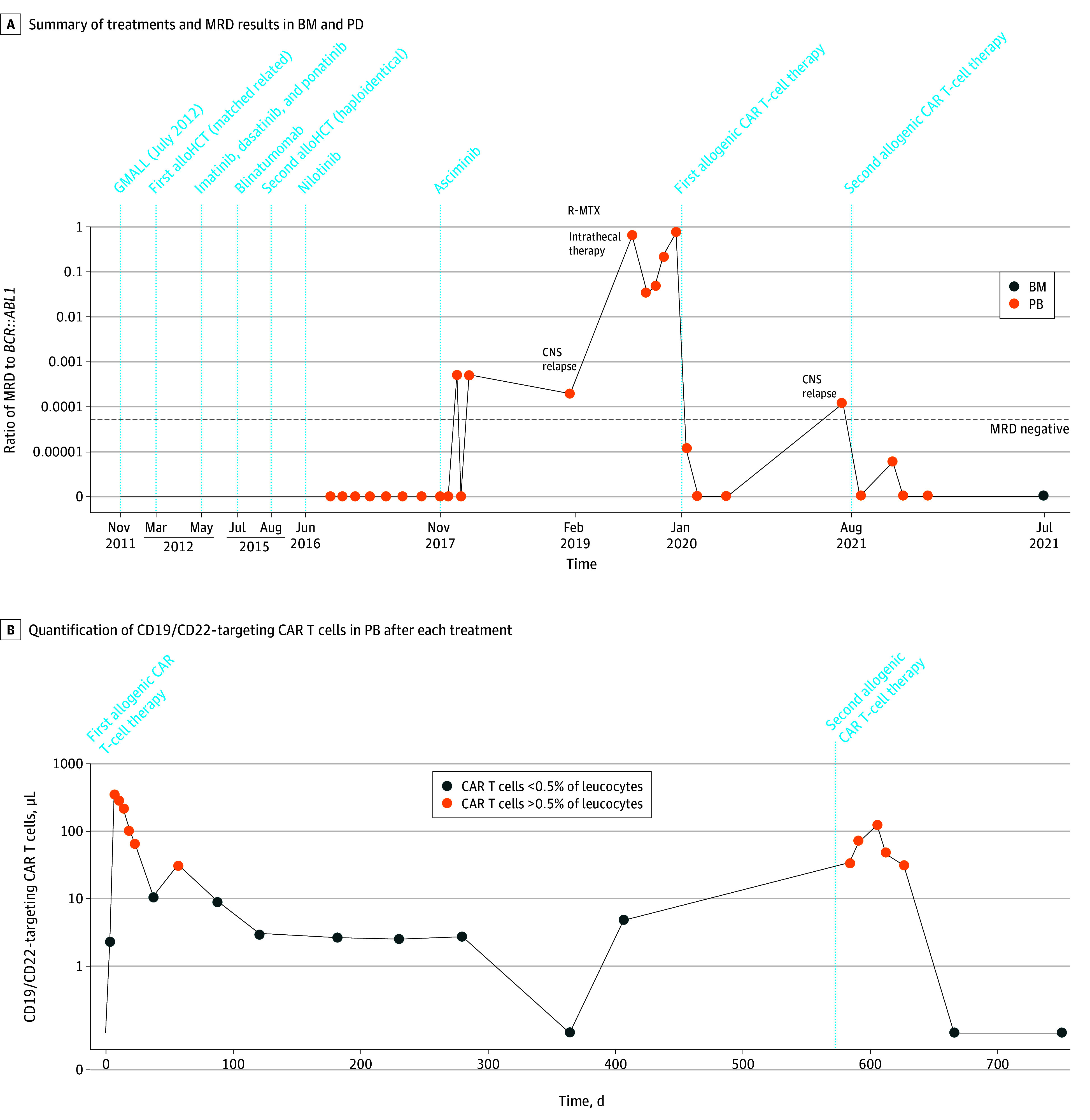
Treatment Response and Chimeric Antigen Receptor (CAR) T-Cell Quantification in Patient 2 A, Summary of treatments and minimal residual disease (MRD) results in bone marrow (BM) and peripheral blood (PB) (molecular *BCR::ABL1:ABL1* ratio along logarithmic scale; MRD values prior to August 2016 are omitted for legibility). AlloHCT indicates allogeneic hematopoietic cell transplant; CNS, central nervous system; GMALL, the German Multicenter Study Group acute lymphoblastic leukemia regimen; R-MTX, rituximab and methotrexate. B, Quantification of CD19/CD22-targeting CAR T cells in PB after each CAR T-cell treatment.

A man in his late 50s with hematologic and central nervous system *BCR::ABL*-positive B-cell ALL relapse after receiving chemotherapy, sibling alloHCT, blinatumomab, haploidentical alloHCT, and multiple tyrosine kinase inhibitors (Figure 2A) received 3 × 10^6^/kg bodyweight patient-derived fresh allogeneic CD19/CD22-targeting CAR T cells, which expanded with cytokine release (Figure 2B) and induced molecular (Figure 2A) and central nervous system remission. Eighteen months later, CD19^+^/CD22^+^ relapse occurred, and the patient was treated with 6 × 10^6^/kg bodyweight cryopreserved CAR T cells, inducing transient neurotoxic effects. Twenty-four months later, the patient showed durable remission and persisting CAR T cells (Figure 2).

## Discussion

In this case series, we report that repeated treatments with locally manufactured allogeneic bispecific humanized CD19/CD22-targeting CAR T cells were feasible and associated with durable remission in 2 patients with relapsed/refractory ALL receiving alloHCT and antibody treatments. Antigen loss was not observed as resistance mechanism after treatment with CD19/CD22 CAR T cells. The use of allogeneic (donor derived) healthy cells potentially improved CAR T-cell functionality without adding toxic effects (eg, graft-vs-host disease).

CD19 or CD22 antibodies can reduce minimal residual disease in relapsed/refractory B-cell ALL,^6^ and autologous CD19/CD22 CAR T cells were previously reported.^3,4^ Outcomes after repeated use of allogeneic CD19/CD22-targeting CAR T cells combined with sequential antibody therapies are, to our knowledge, yet unreported.

A limitation to this study is that data are only from 2 patients. Allogeneic bispecific humanized CAR T-cells show promising results but require prospective testing. Nonetheless, allogeneic humanized CD19/CD22-targeting CAR T-cells may induce durable remission in patients with relapsed/refractory B-cell ALL.

## Notes

Supplement 1.
eMethods

Supplement 2. Data Sharing Statement
