# Supplementary material for: Allogeneic CD19/CD22 CAR T-Cell Therapy for B-Cell Acute Lymphoblastic Leukemia
Source: JAMA Oncol. 2024 Apr 18;10(6):821–4. doi: 10.1001/jamaoncol.2024.0473 (PMC11190796; doi:10.1001/jamaoncol.2024.0473)
Supplement: Supplement 1. — eMethods [file jamaoncol-e240473-s001.pdf]

Supplemental Online Content

Phely L, Hensen L, Faul C, et al. Allogeneic CD19/CD22 CAR T-cell therapy for B-cell acute lymphoblastic leukemia. *JAMA Oncol*. Published online April 18, 2024. doi:10.1001/jamaoncol.2024.0473

eMethods

This supplemental material has been provided by the authors to give readers additional information about their work.

© 2024 Phely L et al. *JAMA Oncol.*

eMethods

Bispecific CD19/CD22 targeting CAR-T-cell manufacturing. Fresh allogeneic mononuclear cells obtained by leukapheresis from patients or the healthy allogeneic donor were transduced with a lentiviral vector encoding a human anti-CD19 scFv M19217-1 and human anti-CD22 scFv 16p17 CAR on a human CD8 hinge and CD8 transmembrane domain, CD3ζ intracellular domain and 4-1BB costimulatory domain to manufacture bispecific CD19/CD22 CAR-T-cells in a 12-day process using an established protocol on a Clinimacs Prodigy cell processing system (Miltenyi Biotec GmbH, Bergisch-Gladbach) in the Good Manufacturing Practice Laboratory at the University Hospital Tübingen.

Lymphodepletion and CRS management: Lymphodepletion with 30mg/m^2 fludarabine on day -5 to -3 and 1000mg/m^2 cyclophosphamide on day -3 was applied prior to the infusion of 3x10E6 (or respectively 6x10E6, as indicated) CD3 positive fresh (or respectively cryopreserved, as indicated) CD19/22 CAR-T-cells/kg on day 0. A prophylactic dose of 4mg/kg Tocilizumab was applied on day 0 prior to CAR-T-cell application, and the patients were monitored for CRS and ICANS as inpatient for at least 10 days post-therapy.

Routine diagnostic work-up and assessment of response to treatment: Minimal residual disease (MRD), interleukin-6 (IL-6) and -2-receptor (IL-2-rec.) measurements, and magnetic resonance imaging (MRI) were part of standard of care. Response rates were assessed 6 weeks, 3 months, 6 months, and at further time-points according to the standards of clinical care. The latest follow-up response assessment was performed at days 968 and 1160 days, respectively, after 1st CAR-T-cell administration. Hematologic response was assessed by cytology, histopathology, flow cytometry of peripheral blood (PB) and bone marrow (BM) samples, and imaging as required. Minimal residual disease (MRD) was assessed in BM and PB samples by quantitative real-time PCR as part of the routine diagnostics in reference laboratories at the University Hospitals Frankfurt and Schleswig-Holstein (UKSH). Complete cytological remission was defined as <5% blasts in bone marrow aspirates and molecular CR was defined as an MRD ratio below 10e-4 in quantitative PCR for BCL-ABL1 transcript or clone-specific-immunoglobulin-rearrangements (IGH V3-21, D3-16 J8).

Flow cytometry quantification of anti-CD19/CD22 CAR-T-cells: Anti-CD19/CD22 CAR-T-cells were quantified among purified PBMCs or fresh anticoagulated blood samples using the CAR detection reagent (Miltenyi Biotec), and non-CAR T cells analyzed after staining with the following mouse anti-human antibodies: CD45-FITC (HI30), CD3-APC (UCHT1), CD4-BV605 (RPA-T4), CD8-BV786 (RPA-T8), CD45RA-PE-Cy7 (L48) and CD27-BV510 (L128), all from BD Biosciences using a BD FACS Lyric Flow Cytometer and the BD FACSuite software.

The patients provided written consent to publication of their data and IRB approval was granted to the study.

© 2024 Phely L et al. *JAMA Oncol.*
