# Supplementary material for: Allogeneic CD19/CD22 CAR T-Cell Therapy for B-Cell Acute Lymphoblastic Leukemia
Source: JAMA Oncol. 2024 Apr 18;10(6):821–4. doi: 10.1001/jamaoncol.2024.0473 (PMC11190796; doi:10.1001/jamaoncol.2024.0473)
Supplement: Supplement 2. — Data Sharing Statement [file jamaoncol-e240473-s002.pdf]

Data Sharing Statement

Phely. Allogeneic CD19/CD22 CAR T-Cell Therapy for B-Cell Acute Lymphoblastic Leukemia. *JAMA Oncol*. Published April 18, 2024. doi:10.1001/jamaoncol.2024.0473

Data
Data available: Yes
Data types: Deidentified participant data
How to access data: All datasets of the current study will be available from the corresponding author on reasonable request (Claudia.Lengerke@med.uni-tuebingen.de)
When available: With publication

Supporting Documents
Document types: None

Additional Information
Who can access the data: researchers whose proposed use of the data has been approved
Types of analyses: for any purpose
Mechanisms of data availability: with investigator support
